# Trends in primary graft dysfunction and early mortality following lung transplantation: A single center experience

**DOI:** 10.1016/j.jhlto.2026.100488

**Published:** 2026-01-15

**Authors:** Yuriko Terada, Michael K. Pasque, Chad A. Witt, Ruben G. Nava, Benjamin D. Kozower, G. Alexander Patterson, Daniel Kreisel, Varun Puri, Ramsey R. Hachem, Tsuyoshi Takahashi

**Affiliations:** a Division of Cardiothoracic Surgery, Department of Surgery, Washington University, Saint Louis, MO; b Division of Pulmonary and Critical Care Medicine, Department of Medicine, Washington University, Saint Louis, MO; c Department of Pathology & Immunology, Washington University, Saint Louis, MO; d Division of Pulmonary Medicine, Department of Internal Medicine, University of Utah School of Medicine, Salt Lake City, UT; e Department of Thoracic Surgery, Kanazawa University, Kanazawa, Ishikawa, Japan

**Keywords:** Lung transplantation, Primary graft dysfunction, Early mortality, Lung allocation score, Trend

## Abstract

**Objectives:**

Although the long-term prognosis after lung transplantation has improved recently, primary graft dysfunction (PGD) remains the major cause of early mortality. The aim of this study was to elucidate trends in PGD incidence and short-term mortality following lung transplantation in the contemporary era.

**Methods:**

We analyzed a single-center database of lung transplantations performed across three periods (Era 1: 2009–2013, Era 2: 2014–2017, and Era 3: 2018–2021). PGD was graded according to the 2016 International Society for Heart and Lung Transplantation definition, and PGD grade 3 within T0–T72 was used as the primary outcome. Trends in PGD incidence, factors associated with PGD, and early mortality rates after lung transplantation were identified.

**Results:**

This study included 856 lung transplants: 277 in Era 1, 296 in Era 2, and 283 in Era 3. PGD grade 3 incidence decreased significantly over time: 35.9% (99 cases) in Era 1, 26.4% (78 cases) in Era 2, and 18.4% (52 cases) in Era 3 (P<0.001). During the study period, the lung allocation score (LAS) and intraoperative cardiopulmonary bypass (CPB) use decreased, whereas the use of intraoperative nitric oxide and extracorporeal membrane oxygenation increased. Logistic multivariate modeling identified era, recipient sex (male), underlying disease, race, and blood transfusion as factors associated with PGD. No significant difference was observed in 30-day hospital mortality across the three eras (2.9%, 1.4%, and 1.4% for Era 1, Era 2, and Era 3, respectively; P=0.313).

**Conclusion:**

This study demonstrated a significant reduction in PGD incidence over time, which coincided with a decrease in LAS and intraoperative CPB use. However, no significant changes were observed in short-term mortality after lung transplantation.

## Introduction

Over the past four decades, lung transplantation has evolved into a well-established treatment option for patients with end-stage pulmonary disease. The number of lung transplantations performed has increased steadily from 11,000 in the 1990s to 34,000 between 2010 and 2018.^1^ According to the International Society for Heart and Lung Transplantation (ISHLT) registry, notable changes in donor and recipient characteristics have been observed across different eras (1992–2000, 2001–2009, and 2010–2018).^1^ The median recipient age increased consistently from 50 years during the 1992–2000 era to 57 years during the 2010–2018 era. In addition, the proportion of male recipients increased in the most recent era, whereas the prevalence of recipients with a history of smoking declined over time. Despite significant improvements in long-term survival among adult lung transplant recipients between 2000 and 2017, attributed to advancements in immunosuppression and surgical techniques,^1^, ^2^, ^3^, ^4^ it remains unclear whether recent changes in donor and recipient characteristics have influenced short-term outcomes compared to earlier eras.

The primary aim of this study was to examine trends in donor and recipient characteristics, as well as short-term outcomes, including the incidence of primary graft dysfunction (PGD) and 30-day mortality over time. The secondary aim was to identify factors associated with PGD development.

## Material and methods

### Data collection and study population

We conducted a retrospective cohort study using a prospectively maintained single-center database of lung transplant donors and recipients at our institution between January 2009 and December 2021. During the study period, 4 multi-organ transplants, 28 re-transplants, 43 single lung transplants, and 2 transplants from donation after circulatory death donors were excluded to maintain a homogeneous cohort of primary bilateral lung transplant recipients. The patients were classified into three eras: Era 1 (2009–2013), Era 2 (2014–2017), and Era 3 (2018–2021). The eras were defined to ensure numerical balance between the groups. The Washington University School of Medicine Institutional Review Board for Human Studies approved the study protocol (ID #202203073). The Institutional Review Board waived the requirement for individual written consent for the publication of the study data because of the retrospective nature of the study.

### Definition

PGD was graded according to the 2016 ISHLT definition.^5^ The PGD grade was assessed at 0, 24, 48, and 72 h post-transplantation, with the highest grade within the first 72 h considered the outcome of interest.

### Statistical analysis

Donor and recipient characteristics, along with operative variables, were summarized using descriptive statistics. Categorical variables are presented as counts (%), and continuous variables are expressed as mean ± standard deviation for normally distributed variables or as median (quartile 1 to quartile 3) for highly skewed data. Comparisons between the groups were conducted using Fisher’s exact test for categorical variables and analysis of variance for continuous variables. The Kaplan—Meier method was used to estimate survival probabilities from the date of lung transplantation to the endpoints of death or censoring.

Univariate logistic regression was performed to identify the risk factors for PGD, and variables with a p-value of <0.05 were included in the multivariate model. Odds ratios (ORs) and 95% confidence intervals (CIs) were evaluated for logistic regression analysis. Temporal trends in PGD incidence were assessed using the Cochran—Armitage trend test. All statistical analyses were performed using SPSS (version 28.0; SPSS Inc.) and JMP Pro 17 (SAS Institute).

## Results

**Figure 1:**
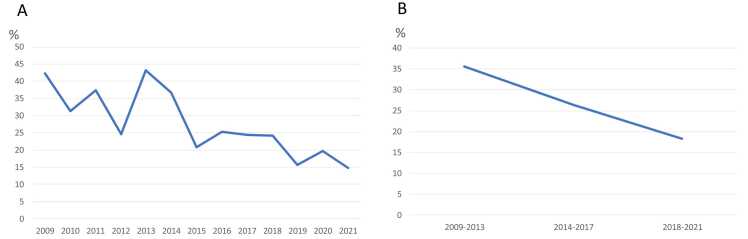
(A) Incidence of PGD grade 3 within 72 h after transplantation stratified by years. (B) Incidence of PGD grade 3 within 72 h after transplantation was 35.9%, 26.4%, and 18.4% in Era 1, Era 2, and Era 3, respectively. Abbreviation: PGD, primary graft dysfunction.

**Figure 2:**
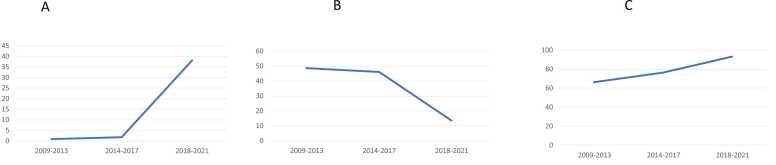
(A) Trends in ECMO use by era. The percentage of intraoperative ECMO use was 0.7%, 1.7%, and 38.2% in Era 1, Era 2, and Era 3, respectively. (B) Trends in CPB use by era. The percentage of intraoperative CPB use was 48.7%, 45.9%, and 13.4% in Era 1, Era 2, and Era 3, respectively. (C) Trends in NO use by era. The percentage of intraoperative NO use was 66.3%, 76.4%, and 92.9% in Era 1, Era 2, and Era 3, respectively. Abbreviations: ECMO, extracorporeal membrane oxygenation; CPB, cardiopulmonary bypass; NO, nitric oxide.

A total of 856 lung transplantations were included in the study and categorized according to era as follows: Era 1 (n=277), Era 2 (n=296), and Era 3 (n=283). PGD incidence significantly decreased over time (35.9% in Era 1, 26.4% in Era 2, and 18.4% in Era 3; Cochran—Armitage test, p<0.001; Figure 1A, B). Donor characteristics, including age, sex, smoking history, and best PaO_2_, did not differ significantly between the eras (Table 1). However, there was a significant decrease in the proportion of white donors (73.3% in Era 1, 74.0% in Era 2, and 67.1% in Era 3) and stroke as the cause of death (37.9% in Era 1, 29.7% in Era 2, and 26.5% in Era 3). Recipient characteristics, including sex, ethnicity, smoking history, and cytomegalovirus mismatch, were consistent across eras. Nonetheless, recipient age increased significantly over time: 56.0 in Era 1, 59.5 in Era 2, and 61.0 years in Era 3 (p<0.001), as did the proportion of recipients with restrictive lung disease (46.2% in Era 1, 54.1% in Era 2, and 54.8% in Era 3). In contrast, the lung allocation score (LAS) decreased (40.4% in Era 1, 39.3% in Era 2, and 37.9% in Era 3; p<0.001), whereas the ischemic time increased significantly (269 min in Era 1, 246 min in Era 2, and 309 min in Era 3; p<0.001). Operative trends also changed over time, with a significant increase in the use of extracorporeal membrane oxygenation (ECMO; Cochran—Armitage test, p<0.001; Figure 2A), and a decrease in the use of cardiopulmonary bypass (CPB; Cochran—Armitage test, p<0.001; Figure 2B). An increase in the use of nitric oxide (NO; Cochran—Armitage test; p<0.001, Figure 2C) was also noted. Despite these changes, neither 30-day hospital mortality nor 1-year mortality differed significantly among the eras. Thirty-day hospital mortality was 2.9% in Era 1, 1.4% in Era 2, and 1.4% in Era 3 (p=0.313; Table 2), and 1-year mortality was 11.6%, 9.5%, and 14.5% in Eras 1, 2, and 3, respectively (p=0.225).

**Table 1:** Baseline Characteristics of Donors and Recipients

Variables	Era 1 (n=277)	Era 2 (n=296)	Era 3 (n=283)	*P* Value
Donor				
Age (years)	35 (24–50)	34 (23–49)	34 (23–48)	0.830
Male	174 (62.8)	170 (57.4)	183 (64.7)	0.177
Ethnicity				0.012
White	203 (73.3)	219 (74.0)	190 (67.1)	
Black	63 (22.7)	59 (19.9)	61 (21.6)	
Others	11 (4.0)	18 (6.1)	32 (11.3)	
Cause of death				0.037
Stroke	105 (37.9)	88 (29.7)	75 (26.5)	
Head trauma	115 (41.5)	125 (42.2)	123 (43.5)	
Anoxia	47 (17.0)	75 (25.3)	73 (25.8)	
Others	10 (3.6)	8 (2.7)	12 (4.2)	
Creatinine (mg/dL)	1.0 (0.8–1.2)	1.0 (0.8–1.3)	1.1 (0.9–1.6)	<0.001
Cigarette use	30 (10.9)	41 (13.9)	21 (7.6)	0.051
Best PaO_2_ (mmHg)	506 (461–559)	524 (473–561)	511 (470–565)	0.131
Non-local donor	120 (43.3)	122 (41.2)	206 (72.8)	<0.001
Recipient				
Age (years)	56 (43–62)	60 (49–66)	61 (54–66)	<0.001
Male	160 (57.8)	171 (57.8)	180 (63.6)	0.261
Ethnicity				0.664
White	258 (93.1)	266 (89.9)	258 (91.2)	
Black	15 (5.4)	22 (7.4)	20 (7.1)	
Others	4 (1.4)	8 (2.7)	5 (1.8)	
Indications for transplant				0.005
Obstructive lung disease	88 (31.8)	84 (28.4)	91 (32.2)	
Pulmonary vascular disease	6 (2.2)	3 (1.0)	12 (4.2)	
Cystic fibrosis	53 (19.1)	47 (15.9)	23 (8.1)	
Restrictive lung disease	128 (46.2)	160 (54.1)	155 (54.8)	
Others	2 (0.7)	2 (0.7)	2 (0.7)	
Cigarette use	146 (52.7)	153 (51.7)	168 (59.4)	0.135
Lung allocation score	40 (34–53)	39 (35–49)	38 (34–46)	0.011
CMV mismatch	116 (41.9)	135 (45.6)	130 (45.9)	0.561
PAH	146 (66.7)	172 (75.4)	191 (78.3)	0.014
Preoperative MV	24 (8.7)	24 (8.1)	11 (3.9)	0.049
Preoperative ECMO	3 (1.1)	6 (2.0)	11 (3.9)	0.082
Lung downsizing	27 (9.7)	22 (7.4)	18 (6.4)	0.313
Total ischemic time (min)	269 (226–327)	246 (199–306)	309 (265–340)	<0.001
Blood transfusion (unit)	2 (0–3)	2 (0–5)	2 (0–5)	<0.001
Intraoperative ECMO	2 (0.7)	5 (1.7)	108 (38.2)	<0.001
Intraoperative CPB	135 (48.7)	136 (45.9)	38 (13.4)	<0.001
Intraoperative NO	183 (66.3)	226 (76.4)	261 (92.9)	<0.001
Postoperative ECMO	7 (2.5)	13 (4.4)	18 (6.4)	0.088

Abbreviations: CMV, cytomegalovirus; CPB, cardiopulmonary bypass; ECMO, extracorporeal membrane oxygenation; MV, mechanical ventilation; NO, nitric oxide; PAH, pulmonary arterial hypertension; PaO_2_, partial pressure of oxygen

**Table 2:** Postoperative Outcomes in Recipients

Variables	Era 1 (n=277)	Era 2 (n=296)	Era 3 (n=283)	*P* Value
Tracheostomy	55 (19.9)	74 (25.0)	54 (19.1)	0.167
PGD grade ≧ 3 (T0-T72)	99 (35.7)	78 (26.4)	52 (18.4)	<0.001
30-day hospital mortality	8 (2.9)	4 (1.4)	4 (1.4)	0.313
Length of hospital stay (days)	15 (11–23)	16 (12–28)	17 (12–31)	0.006

PGD, primary graft dysfunction

Logistic multivariate modeling identified several factors that were significantly associated with PGD. The era of transplantation was a significant factor (p<0.001), with a lower risk of PGD observed in Era 2 than in Era 1 (odds ratio [OR], 0.509; confidence interval [CI], 0.338–0.766; p=0.001) and in Era 3 than in Era 1 (OR, 0.385; CI, 0.238–0.620; p<0.001). Male recipients demonstrated a reduced risk of developing PGD (OR, 0.630; CI, 0.442–0.897; p=0.011). Underlying disease was also associated with PGD risk, with pulmonary vascular disease showing a higher risk than obstructive lung disease (OR, 3.872; CI, 1.406–10.666; p=0.009). Restrictive lung disease was also associated with an increased PGD risk compared with obstructive lung disease (OR, 2.487; CI, 1.553–3.983; p<0.001). Race was also a significant factor (p=0.012), with black recipients having a higher risk of developing PGD than white recipients (OR, 2.033; CI, 1.082–3.82; p=0.027). The operative factors associated with PGD included the use of CPB (OR, 1.480; CI, 1.002–2.185; p=0.049) and blood transfusion (OR, 1.106; CI, 1.058–1.157; p<0.001) (Table 3).

**Table 3:** Univariate and Multivariate Logistic Regression Analyses of Risk Factors for PGD

Variables	Univariate Analysis	*P* Value	Multivariate Analysis	*P* Value
	OR (95% CI)		OR (95% CI)	
Era 1 (2009–2013)	Reference		Reference	
Era 2 (2014–2017)	0.640 (0.448–0.914)	0.014	0.509 (0.338–0.766)	0.001
Era 3 (2018–2021)	0.404 (0.274–0.596)	<0.001	0.385 (0.238–0.620)	<0.001
Donor age	1.006 (0.997–1.016)	0.203		
Donor male	1.006 (0.737–1.373)	0.972		
Donor ethnicity				
White	Reference			
Black	0.934 (0.641–1.361)	0.723		
Others	1.069 (0.593–1.926)	0.825		
Cause of death				
Stroke	Reference			
Head trauma	1.068 (0.749–1.522)	0.716		
Anoxia	0.964 (0.635–1.465)	0.864		
Others	0.436 (0.147–1.295)	0.135		
Donor cigarette use	1.149 (0.713–1.851)	0.569		
Best PaO_2_	0.999 (0.997–1.002)	0.592		
Distant donor	1.059 (0.782–1.453)	0.710		
Recipient age	0.998 (0.997–0.999)	0.032	0.989 (0.972–1.006)	0.213
Recipient male	0.643 (0.474–0.873)	<0.001	0.630 (0.442–0.897)	0.011
Ethnicity				
White	Reference		Reference	
Black	2.628 (1.525–4.528)	<0.001	2.033 (1.082–3.820)	0.027
Others	0.626 (0.178–2.200)	0.465	0.252 (0.059–1.082)	0.064
Indications for transplant				
Obstructive lung disease	Reference		Reference	
Pulmonary vascular disease	4.762 (1.902–11.922)	<0.001	3.872 (1.406–10.666)	0.009
Cystic fibrosis	1.690 (0.997–2.864)	0.051	0.915 (0.488–1.869)	0.808
Restrictive lung disease	2.531 (1.722–3.721)	<0.001	2.487 (1.553–3.983)	<0.001
Others	5.238 (1.022–26.841)	0.047	1.416 (0.209–9.596)	0.721
Recipient cigarette use	0.807 (0.595–1.092)	0.165		
Lung allocation score	1.028 (1.019–1.036)	<0.001	1.006 (0.994–1.018)	0.341
CMV mismatch	1.009 (0.744–1.368)	0.954		
PAH	1.117 (0.758–1.645)	0.576		
Preoperative MV	2.980 (1.738–5.109)	<0.001	1.894 (0.941–3.814)	0.074
Preoperative ECMO	2.283 (0.934–5.585)	0.070		
Lung downsizing	1.088 (0.625–1.893)	0.076		
Total ischemic time	1.003 (1.000–1.005)	0.030	1.002 (0.999–1.004)	0.237
Blood transfusion	1.122 (1.079–1.167)	<0.001	1.106 (1.058–1.157)	<0.001
Intraoperative ECMO	0.827 (0.521–1.311)	0.418		
Intraoperative CPB	3.054 (2.234–4.176)	<0.001	1.480 (1.002–2.185)	0.049
Intraoperative NO	1.009 (0.697–1.459)	0.963		

Abbreviations: CI, confidence interval; CMV, cytomegalovirus; CPB, cardiopulmonary bypass; ECMO, extracorporeal membrane oxygenation; MV, mechanical ventilation; NO, nitric oxide; OR, odds ratio; PAH, pulmonary arterial hypertension; PaO_2_, partial pressure of oxygen.

## Discussion

In this study, we report an improvement in PGD incidence after lung transplantation in recent eras (2014–2017 and 2018–2021) compared with that in the early era (2009–2013). Over the study period, we observed several changes in the donor and recipient demographic characteristics. Specifically, there was an increase in head trauma as the donor cause of death, recipient age, and prevalence of restrictive lung disease, whereas LAS and intraoperative use of CPB decreased. These changes occurred in parallel with an observed improvement in PGD incidence. Our multivariate analysis showed that lung transplants performed in the recent era were independently associated with a significantly lower risk of PGD, regardless of other contributing risk factors. Although transplant era remained independently associated with PGD in multivariable analysis, era likely serves as a proxy for multiple concurrent changes in recipient characteristics and perioperative management over time rather than a single causal exposure. Accordingly, the association between era and PGD should be interpreted cautiously and viewed as reflecting cumulative temporal improvements in practice and case-mix.

In a prospective multicenter cohort study conducted between 2011 and 2018, Cantu et al. reported an overall incidence of PGD grade 3 of 25.7%, with a significant increase from 14.3% in 2011 to 38.2% in 2018.^6^ They also demonstrated that the median LAS significantly increased from 38.0 to 47.7 over the same period, and that a higher LAS was associated with an increased incidence of PGD. The same group previously reported a PGD incidence of 16.8% in a cohort enrolled between 2002 and 2010.^7^ The authors speculated that this increasing trend in PGD grade 3 incidence may be attributed to the growing proportion of sicker patients on the transplant waitlist, characterized by a combination of restrictive lung disease, pulmonary hypertension, and obesity.^6^ In contrast, our study demonstrated a decreasing trend in PGD incidence and LAS in recent years. Although the exact reason for the downward trend in LAS over time remains unclear, it may have contributed to the observed improvement in PGD outcomes in this study.

The intraoperative use of CPB has been identified as an independent risk factor for PGD.^7^, ^8^, ^9^ This association may be attributed to hemodynamic instability observed during transplantation, which is linked to an increased risk of PGD. A recent multicenter international study reported that patients undergoing lung transplantation with CPB had a higher risk of PGD grade 3 than those managed with veno-arterial (VA) ECMO or off-pump procedures.^10^ VA-ECMO is increasingly being preferred at high-volume centers as an alternative to CPB for intraoperative cardiopulmonary support,^11^ particularly during double-lung transplantation. At our institution, VA-ECMO has been routinely selected as the primary method for cardiopulmonary support, replacing CPB since 2018. A meta-analysis further demonstrated that VA-ECMO support was associated with reduced PGD incidence, bleeding complications, and renal failure compared with conventional CPB.^11^

PGD is a leading cause of short-term morbidity and mortality after lung transplantation, with a reported mortality rate of up to 40%.^12^, ^13^ Although no statistically significant improvement was observed in short-term prognosis across the eras, our study demonstrated a downward trend in 30-day hospital mortality in the two most recent eras compared to that in the early era. This trend may be partly explained by the relatively small number of patients in our cohort who died within 30 days of lung transplantation during the study period.

Despite the observed reduction in severe PGD incidence over time, the length of hospital stay modestly increased across eras. This apparent discrepancy likely reflects changes in recipient case-mix rather than worsening early graft function. In particular, recipients in the more recent eras were older and more frequently transplanted for restrictive lung disease and pulmonary hypertension, clinical profiles that are often associated with prolonged postoperative recovery even in the absence of severe PGD. In addition, advances in perioperative management—including increased use of intraoperative ECMO and nitric oxide—may have improved early survival among higher-risk recipients who previously might have experienced early mortality. As a result, these patients may survive the immediate postoperative period but require longer hospitalization for recovery and rehabilitation. Finally, institutional changes in postoperative care pathways, discharge planning, and rehabilitation practices over time may also have contributed to longer hospital stays in more recent eras. Taken together, the increasing length of stay likely reflects evolving recipient complexity and improved survival rather than a deterioration in early allograft outcomes.

This study had some limitations. First, this was a retrospective, single-center, observational study, which may have limited the generalizability of the findings. Second, we did not account for the experience level of lung transplant surgeons or specific technical aspects of the surgical procedures, both of which could have influenced the outcomes. Finally, there may have been other potential risk factors of PGD that were not included in our analysis, which could have affected the results.

## Conclusions

We observed a decreasing trend in severe PGD (PGD grade 3), defined as the highest PGD grade within the first 72 h after transplantation (T0-T72), incidence following lung transplantation. This improvement in PGD incidence in recent years is likely multifactorial; however, the reduction in recipients’ LAS and decreased use of CPB may have contributed significantly to this decline.

## Funding statement

none.

## Declaration of Competing Interest

The authors declare that they have no known competing financial interests or personal relationships that could have appeared to influence the work reported in this paper.
